# YOLOv8n-RF: A Dynamic Remote Control Finger Recognition Method for Suppressing False Detection

**DOI:** 10.3390/s25092768

**Published:** 2025-04-27

**Authors:** Yawen Wang, Gaofeng Wang, Yining Yao

**Affiliations:** College of Computer Science and Engineering, Xi’an Technological University, Xi’an 710021, China; wgaofeng25@163.com (G.W.); jay2583@163.com (Y.Y.)

**Keywords:** multi-scale features, YOLOv8n-RF, attention mechanism, remote-controlled finger recognition, deep learning

## Abstract

Gesture interaction is one of the novel human-computer interaction methods for smart TVs. Addressing the issues of false detection and high detection costs in gesture recognition algorithms for gesture interaction, this paper proposes the YOLOv8n-Remote Finger (YOLOv8n-RF) algorithm for dynamic remote control finger detection. This algorithm utilizes the CRVB-DSConvEMA module in the feature extraction network, adopts the SPPF-DSConvEMA module in the downsampling process, and introduces BiFPN in the Neck layer. Experiments conducted on the self-made Remote Finger dataset and the public HaGRID dataset demonstrated that, compared to the YOLOv8n algorithm, the proposed YOLOv8n-RF algorithm achieved an improvement in mean Average Precision (mAP) by 1.23% and 0.84%, respectively. Additionally, the model size was reduced by 2.49 M, the GFLOPs were decreased by 1.7, and the false detection rate was lowered by 22%. The YOLOv8n-RF algorithm meets the requirements of low cost and low complexity, which contributes to reducing false control operations on smart TVs.

## 1. Introduction

Through the integration of artificial intelligence technology, smart TVs have expanded a number of functions, including voice control and gesture recognition. Although gesture interaction technology has great potential and is intuitive and natural, its popularity is not as high as voice interaction, and the problem of false detection in gesture interaction may lead to false control, thus affecting user experience. Therefore, it is of great significance to study gesture interaction of smart TVs, reduce gesture false detection rate, and achieve universal applicability.

Regarding the research on gesture interaction in smart TVs, Sang-Heon Lee’steam [1] proposed a gesture recognition method based on a single camera. This method integrates the Adaboost algorithm, the KLT (Kanade-Lucas-Tomasi) tracker, and the SVM algorithm, achieving a high recognition rate for five types of gestures. With further research, Lian S’steam [2] addressed the issue of power consumption in gesture recognition modules and camera devices by proposing a solution aimed at reducing the computational cost and energy consumption of smart TV systems, thereby promoting the practical application of gesture interaction technology in smart TVs. Meanwhile, J. Ducloux’s team [3] took a different approach and designed an accelerator-based gesture recognition system. This system identifies gesture commands by pressing a button on the remote control and converts the gesture into a control command upon button release. This method not only simplifies user operations but also improves the accuracy and efficiency of gesture recognition. Neha Dawar’s team [4] introduced depth cameras and wearable inertial sensor devices, further expanding the application scenarios and possibilities of gesture detection. Huiyue Wu’s team [5] proposed a TV control system based on a combination of static and dynamic gesture models, mapping nineteen types of custom gestures to specific operational functions, thereby achieving a more flexible and intuitive gesture interaction experience.

In recent years, Yi Zhang’s team [6] has utilized Doppler radar sensors and a machine learning-based Doppler signal classification algorithm to achieve efficient recognition of predefined gestures. This research provides new ideas and methods for smart TV gesture interaction technology. Additionally, Zimo Liao’s team [7] proposed a solution that supports gesture control without hardware modifications. This solution enables mid-air gesture recognition based on screen and ambient light sensors, further reducing the implementation difficulty and cost of smart TV gesture interaction technology. Bayan Ibrahimm Alabdullah’s team [8] proposed a TV control model based on a recurrent neural network, which improves the accuracy of smart TV gesture recognition by computing skeletal features. Yuri Suzuki’s team [9] developed a gesture recognition system using a mat embedded with optical reflection sensors and accelerometers as an interface for controlling household appliances. Huijing Wang’s team [10] combined convolutional neural networks (CNNs) with the MPCNN transfer learning strategy to propose a novel smart TV control system.

Deep learning technology has significantly contributed to the advancement of gesture detection. Inspired by the performance of CNNs, Jaya Prakash Sahoo’s team [11] proposed an end-to-end fine-tuning method. This method transfers the weights of a pre-trained CNN to a network trained on the target dataset. During this process, the weights and biases of the pre-trained CNN model are updated after each iteration on the target dataset. Md. Ahasan Atick Faisal’s team [12] developed a low-cost wireless data glove that combines flexible sensors and IMUs, and proposed a novel prediction method based on deep learning that converts one-dimensional time series signals into two-dimensional images by introducing spatial projection to achieve dynamic gesture recognition. In addition, the proposed parallel path neural network architecture has a better ability to extract features from multi-modal data than traditional architectures. Due to the challenges of gesture recognition in complex backgrounds, Weina Zhou’s team [13] proposed an encoder-decoder structure and integrated DRN with ASPP as the encoder module to effectively address the problem of gesture segmentation. To address the challenge that RNN, TCN, and 3DCNN struggle to effectively extract spatiotemporal features and classify noisy and complex skeleton sequences, Adam A. Q. Mohammed’s team [14] proposed a deep integrated gesture recognition network (MMEGRN) to leverage various capabilities in extracting and classifying skeleton sequences. Qi Sun’s team [15] achieved high-precision gesture recognition by optimizing the convolutional neural network (CNN). Abu Saleh Musa Miah’steam [16] utilized graphical models and general deep learning networks to extract effective gesture features. As the exploration of transformer models in gesture recognition tasks remains limited, Mallika Garg’s team [17] proposed a novel GestFormer architecture for dynamic gesture recognition. Fo Hu’s team [18] proposed the Spatio-Temporal Fusion Network with Brain Region Partitioning Strategy (STFN-BRPS), where the Recurrent Multi-scale Convolution Module (RMSCM) combines multi-scale convolution and CNN-BiLSTM for temporal feature extraction, the Dynamic Graph Convolution Module (DGCM) establishes electrode connections using functional partitioning strategy, and the Feature Fusion Module (FFM) with channel attention adaptively weights spatio-temporal features, improving detection accuracy and robustness.

YOLO is an algorithm that balances real-time performance with high accuracy. To solve the problem of excessive parameters and high complexity in this algorithm, Dan Zhao’s team [19] developed a kiwifruit detection algorithm based on Faster-YOLOv8n. Their approach replaces all C2f modules in the YOLOv8n model with C2f-Faster modules, where feature maps are processed using partial convolution (PConv) and pointwise convolution (PWConv) while maintaining residual connections. To address the issues of low detection accuracy, missed detections, false positives, and excessive model parameters, Xiang Li’s team [20] proposed the 2SC-YOLOv8n steel defect detection model. This model integrates the Context Guided Block module and Shuffle Attention mechanism into the Backbone of YOLOv8n and adds the SENetV2 attention module to the Neck layer.

In summary, existing research often requires multiple peripheral devices or wearable equipment, leading to additional costs. Furthermore, YOLOv8n suffers from issues of false detections and model complexity. Therefore, the current research focus is on improving the accuracy of monocular camera gesture detection while maintaining a lightweight model and minimizing false detections that result in erroneous control. The contributions of this paper are as follows:

1. The CRVB-DSConvEMA module is used to replace the C2f module, enabling the model to adaptively focus on local details in prominent gestures based on the input feature map. Additionally, it enhances features across multiple scales, different channels, and spatial information.
2. The SPPF-DSConvEMA module is used to replace the SPPF module. By reconstructing partial channels and evenly distributing spatial semantics among sub-features, it preserves multi-angle information while improving computational efficiency. This approach further reduces false detections without increasing the model’s complexity.
3. The BiFPN module is adopted in the Neck layer of YOLOv8n, enabling the model to achieve greater performance gains with minimal computational cost. While ensuring the effectiveness and accuracy of the previous two steps, this approach further reduces the model’s complexity.

## 2. Materials and Methods

### 2.1. Materials

#### 2.1.1. RemoteFinger Dataset

**Figure 1:**
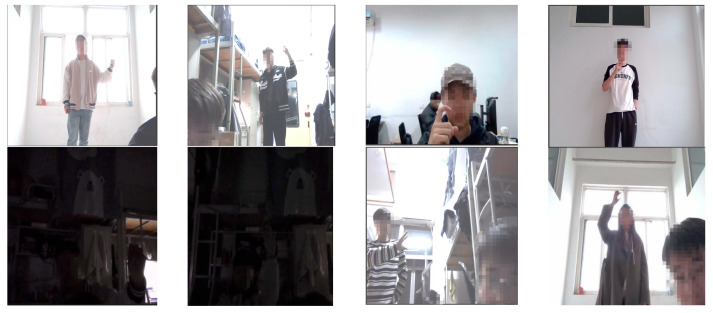
RemoteFinger Dataset Sample Images.

This study, set in the research context of gesture interaction with smart TVs, aims to generate gesture commands using specific hand gestures. Therefore, data collection was conducted with 10 participants, capturing three types of dynamic gesture commands. Utilizing OpenCV, the recorded videos were processed frame by frame, producing a total of 6300 images, which were manually annotated to form the Remote Finger 1.0 dataset. However, after training and testing with YOLOv8, analysis of various performance metrics revealed that the dataset had minimal feature differences between some categories. This led to the misclassification of certain gestures as others. To enhance the distinctiveness of the data, characteristics of the HaGRID dataset were referenced. The original 6300 images were manually filtered to remove adjacent frames with identical hand gestures, ensuring that each image contains a distinct gesture, and additional samples were collected under varying conditions, including different environments, lighting conditions, subjects, and distances between the camera and the hand. Furthermore, data augmentation techniques were applied to enrich the dataset. Sample images are shown in Figure 1, and detailed dataset information is provided in Table 1.

In Table 1, Remote Finger1.0 refers to the initial version of the collected data, while Remote Finger represents the final dataset, which is also the one used in the following experiments.

**Figure 2:**
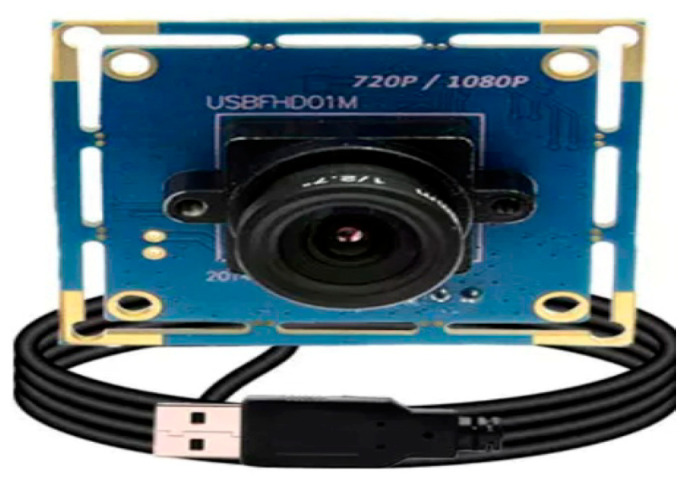
Image sensor device.

Figure 2 shows the gesture image acquisition device, which has a maximum capture resolution of 1920 × 1800. To meet the model’s input requirements, we adjusted the resolution to 640 × 640 during data acquisition.

#### 2.1.2. HaGRID Dataset

Since this dataset is particularly large, with a vast number of images for each category, manual filtering and annotation were performed. The detailed dataset information is provided in Table 2.

#### 2.1.3. Data Augmentation

**Figure 3:**
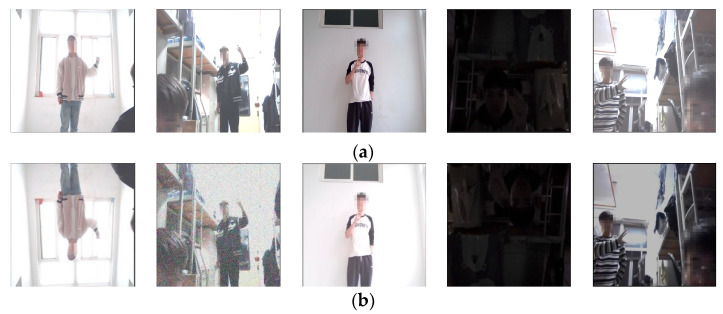
Data Augmentation Sample Images. (**a**) Original Image; (**b**) Transformed Image, Corresponding to Flipping, Gaussian Noise, Brightening, Rotation, and Darkening operations.

Since the custom dataset underwent manual filtering after collection, the total number of samples was slightly reduced. To expand the dataset and improve the quality of certain samples, data augmentation techniques such as mirroring, brightness adjustment (both increasing and decreasing), and adding Gaussian noise were applied. The processed results are shown in Figure 3.

The code logic for adding Gaussian noise to the image is as follows (Algorithm 1):
**Algorithm 1.** Gaussian Noise algorithmI: input image of size W×H×C μ: noise mean (default 0) σ: noise standard deviation (controls intensity) AddGaussianNoise $\left(I,\mu ,\sigma \right)$:            $I_{noise}=copy\left(I\right)$   # Initialize output image            for each pixel coordinate $\left(x,y\right)$:                  for each channel $c\in \left\{R,G,B\right\}$:                          $\gamma \sim N\left(\mu ,\sigma^{2}\right)$   # Sample from Gaussian distribution                          $I_{noise}\left(x,y,c\right)=I\left(x,y,c\right)+\gamma$                          $I_{noise}=ClipToRange\left(I_{noise},\;0,\;255\right)$                  end for              end for              return $I_{noise}$


The formula for vertical flipping is shown in (1), the formula for brightness change is shown in (2), and the formula for rotation is shown in (3).

(1) $$I_{flip}\left(x,y,c\right)=I\left(x,H-1-y,c\right)$$

(2) $$I_{adj}\left(x,y,c\right)=ClipToRange\left(\alpha \cdot I\left(x,y,c\right)+\beta ,0,1\right)$$

(3) $$\left\{\begin{array}{c} x^{\prime }=\left(x-x_{c}\right)\cos\theta -\left(y-y_{c}\right)\sin\theta +x_{c}\\ y^{\prime }=\left(x-x_{c}\right)\sin\theta -\left(y-y_{c}\right)\cos\theta +y_{c} \end{array}\right.$$

In Equations (1)–(3), $I_{flip}$ is the flipped image, $I\in R^{W\times H\times C}$ is the original image, x is the column coordinate, y is the row coordinate, c represents the channel, $I_{adj}$ is the brightness-adjusted image, ClipToRang function ensures the result stays within the valid range, α>0 is the contrast coefficient, β is the brightness offset, $\left(x_{c},y_{c}\right)$ are the rotation center coordinates, $\left(x^{\prime },y^{\prime }\right)$ are the coordinates of the rotated pixel, θ is the rotation angle.

### 2.2. Methods

#### 2.2.1. Network Architecture

Combining the research background, the proposed method in this paper is based on YOLOv8n as the baseline model. This model consists of three main components: the feature extraction backbone (Backbone), the feature enhancement module (Neck), and the detection head (Head). The Backbone is responsible for extracting fundamental features from images and includes two key components: the C2f module and the SPPF module. The C2f module integrates multiple layers through convolutional fusion, facilitating the capture of multi-scale and contextual information. This design enhances feature representation and detection accuracy by capturing semantic information and contextual relationships in images, thereby improving the model’s generalization ability. Additionally, it optimizes computational efficiency by simplifying the convolutional process and stacking multiple layers. The SPPF module consists of convolution and pooling operations, which concatenate feature maps of different scales along the channel dimension. This approach enables the extraction of multi-scale feature information. In the Neck layer, cross-stage feature transmission is employed to facilitate the transfer and fusion of feature information across different levels. The Head layer plays a crucial role in detection by separating classification and localization tasks. This layered design minimizes interference between tasks, making the optimization process more independent.

**Figure 4:**
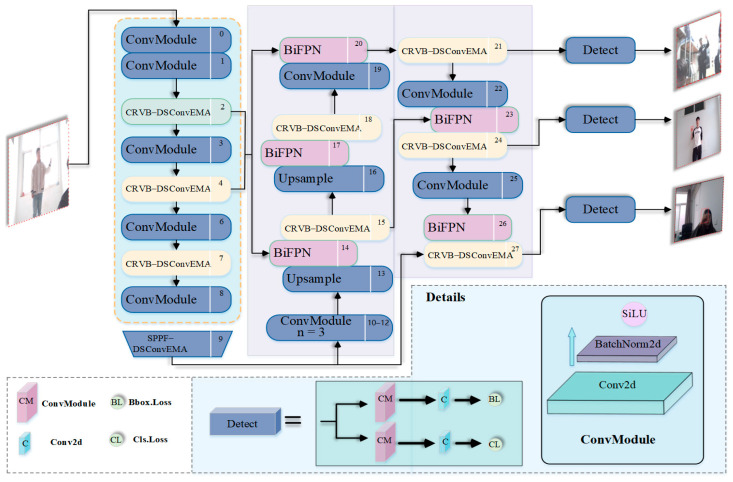
YOLOv8n-RF Network Architecture Diagram.

Therefore, this paper improves the model by focusing on the C2f module, SPPF module, and Neck layer, aiming to reduce false detections while maintaining other performance metrics and lowering model complexity. The proposed YOLOv8n-RF dynamic remote finger detection network architecture, designed to suppress false detections, is illustrated in Figure 4.

This model improves the Backbone of YOLOv8n, which heavily relies on the C2f module to utilize multi-scale features and contextual information, thereby enhancing detection accuracy. However, stacking multiple C2f modules may introduce redundant information, and the standard convolutions used in this module mainly process local features, potentially limiting their effectiveness in capturing global feature relationships. To address these issues, the C2f module is replaced with the CRVB-DSConvEMA module, which integrates the RepViT Block from RepViT [21], DSConv, and EMA [22] to overcome the limitations of C2f. Additionally, the SPPF module, which frequently performs downsampling, may lead to the loss of certain positional information, reducing image resolution. This makes it more challenging for the network to precisely locate objects, thereby increasing the difficulty of target recognition. To mitigate this, the SPPF module is replaced with the SPPF-DSConvEMA module, which avoids dimensionality reduction by reconstructing partial channels and evenly distributing spatial semantics across sub-features. Regarding the Neck layer, which includes convolution, upsampling, and concatenation operations, the increased model complexity needs to be addressed. To achieve this, the BiFPN [23] module is introduced, which provides significant performance improvements with minimal computational cost. Ultimately, these optimizations ensure that the model maintains low complexity while effectively reducing false detections.

#### 2.2.2. CRVB-DSConvEMA Module

To reduce false detections, enhance the network’s ability to recognize subtle differences in dynamic gesture information, and minimize misinformation during inference, the proposed CRVB-DSConvEMA module integrates RepViT Block, DSConv, and EMA.

**Figure 5:**
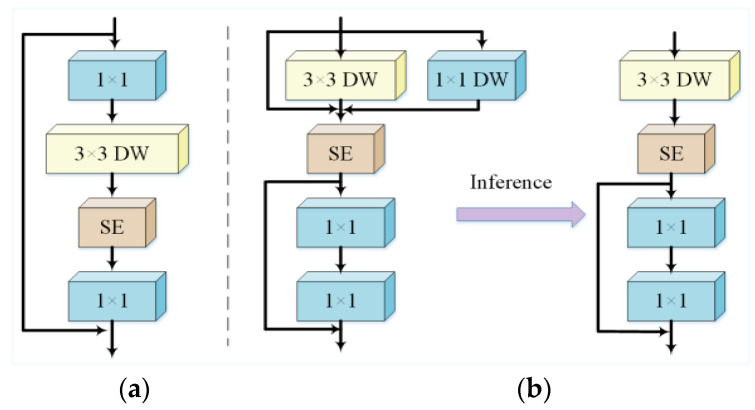
MobileNetBlock and RepViT Block Structure Diagram. (**a**) MobileNetBlock; (**b**) RepViT Block.

Among them, RepViT (Revisiting Mobile CNN From ViT Perspective) [21] is a novel lightweight CNN series that re-examines and integrates lightweight CNNs from a ViT (Vision Transformer) perspective. By leveraging the architecture and optimization strategies of lightweight ViTs, it improves model performance and efficiency while maintaining low computational complexity. Its core structure, RepViT Block, transforms MobileNetV3 by separating the token mixer and channel mixer [24]. The specific structure of this module is shown in Figure 5.

As shown in Figure 5a, the MobileNetBlock consists of a 1×1 convolution block, a depthwise convolution, and a 1×1 projection layer. The 1×1 expansion convolution layer and the 3×3 depthwise convolution enable information exchange between channels, while the depthwise convolution is responsible for spatial feature fusion. These two operations correspond to the channel mixer and token mixer, respectively. As shown in Figure 5b, the RepViT Block shifts the 3×3 depthwise convolution upward and employs structural re-parameterization, introducing a multi-branch topology during training to enhance performance. During inference, the multi-branch structure is merged into a single-branch structure, eliminating the additional computational and storage costs associated with multi-branch configurations.

**Figure 6:**
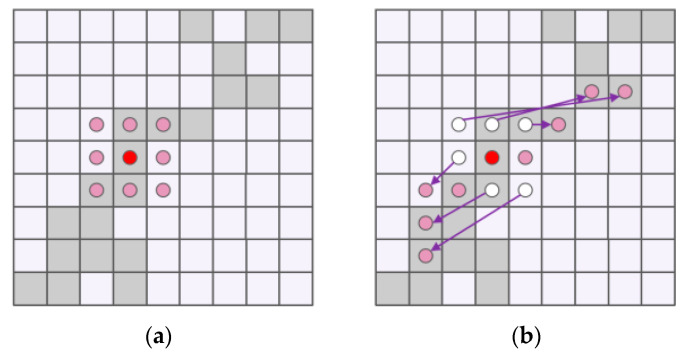
Comparison Diagram of Dynamic Snake Convolution and Standard Convolution. (**a**) Standard Convolution; (**b**) Dynamic Snake Convolution.

Dynamic Snake Convolution (DSConv) [25] introduces deformable offsets, enabling the learning of displacement values and adaptive adjustment of kernel sizes to accommodate object shape variations. It also employs an iterative strategy, sequentially selecting the next position of the target to be processed for observation. This ensures continuity of attention, supplements key features from multiple perspectives, and achieves efficient multi-view feature fusion. A comparison between Dynamic Snake Convolution and standard convolution is shown in Figure 6.

DSConv is a combination of traditional convolution and deformable convolution. For 2D convolution, assuming the convolution kernel size is 3×3 with the center coordinate represented as $\left(x_{i},y_{i}\right)$, a standard convolution can be expressed as:

(4) $$K=\left\{\left(x-1,y-1\right),\left(x-1,y\right),\ldots ,\left(x-1,y+1\right)\right\}$$

To prevent the receptive field from drifting outside the target and to achieve better focus, a standard convolution kernel offset Δ is introduced, adding constraints to the standard convolution kernel in both the *x*-axis and *y*-axis directions.

(5) $$K_{i\pm c}=\left\{\begin{array}{c} \left(x_{i+c},y_{i+c}\right)=\left(x_{i}+c,y_{i}+\sum_{i}^{i+c}\Delta y\right)\\ \left(x_{i-c},y_{i-c}\right)=\left(x_{i}-c,y_{i}+\sum_{i-c}^{i}\Delta y\right) \end{array}\right.$$

(6) $$K_{j\pm c}=\left\{\begin{array}{c} \left(x_{j+c},y_{j+c}\right)=\left(x_{i}+\sum_{j}^{j+c}\Delta x,y_{i}+c\right)\\ \left(x_{j-c},y_{j-c}\right)=\left(x_{j}+\sum_{j-c}^{j}\Delta x,y_{i}-c\right) \end{array}\right.$$

Among them, ∑ represents the accumulation of the offset. However, since the offset Δ is generally a decimal, bilinear interpolation is used to process its integer coordinates, namely:

(7) $$K=\sum_{K^{\prime }}B\left(K^{\prime },K\right)\cdot K^{\prime }$$

In the expression, K represents the decimal position in expressions (5) and (6), and B represents bilinear interpolation, which can be decomposed into two one-dimensional interpolation kernels:

(8) $$B\left(K,K\prime \right)=b\left(K_{x},K_{x}^{\prime }\right)\cdot b\left(K_{y},K_{y}^{\prime }\right)$$

**Figure 7:**
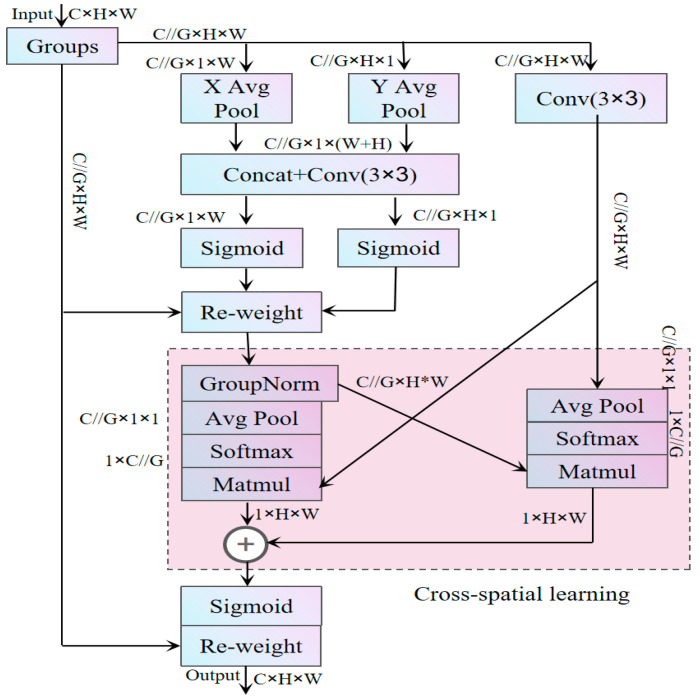
EMA structure diagram.

To enhance feature extraction capability, a high-efficiency multi-scale attention (EMA) mechanism [22] is introduced. This mechanism dynamically adjusts the weights in the feature map based on the importance of each region. EMA achieves this by reorganizing a portion of the channels into the batch dimension and grouping the channel dimension into multiple sub-features, ensuring a uniform distribution of spatial semantic features within each feature group. This approach helps maintain channel-related information while minimizing computational overhead, allowing the module to focus on task-critical regions and improving its adaptability in complex scenarios. Its structure is shown in Figure 7.

In Figure 7, let the input tensor be $x\in R^{C\times H\times W}$, with C as the number of input channels, H and W as the spatial dimensions of the input features. Therefore, the global information one-dimensional average pooling along the horizontal dimension of C at height H is given by:

(9) $$Z_{C}^{H}\left(H\right)=\frac{1}{W}\sum_{0\leq i\leq W}x_{c}\left(H,i\right)$$

Similarly, the global information one-dimensional average pooling along the horizontal dimension of C at width W is given by:

(10) $$Z_{C}^{W}\left(W\right)=\frac{1}{H}\sum_{0\leq i\leq H}x_{c}\left(i,W\right)$$

Finally, the formula for the two-dimensional global pooling operation is:

(11) $$Z_{C}=\frac{1}{H\times W}\sum_{j}^{H}\sum_{i}^{W}x_{C}\left(i,j\right)$$

GroupNorm (Group Normalization) used in Figure 7 is applied to normalize the grouped features, ensuring sufficient feature distribution within each group. It divides C into G  groups, with each group containing C/G channels, and normalizes the groups. The specific calculation formula is:

(12) $$GroupNorm\left(X\right)=\gamma \cdot \frac{X-\mu }{\sqrt{\sigma^{2}+\varepsilon }}+\beta$$

In the expression (12), μ and σ represent the mean and variance of each group’s features, $\gamma \in R^{C}$ denotes the scaling parameter; and $\beta \in R^{C}$ is the shifting parameter.

**Figure 8:**
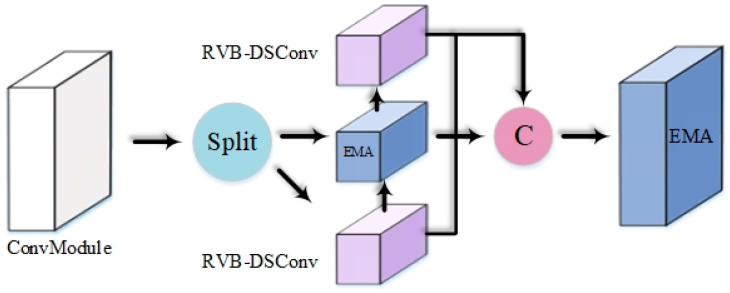
CRVB-DSConvEMA structure diagram.

**Figure 9:**
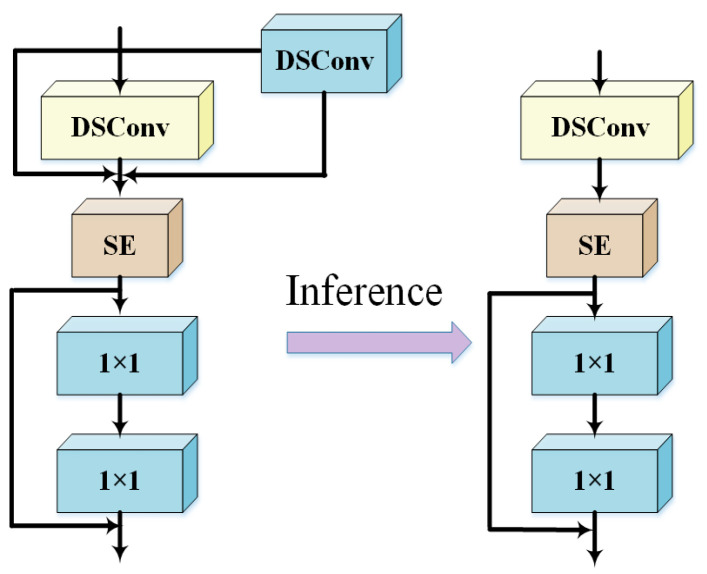
RVB-DSConv Structure Diagram.

In this study, the depthwise convolution component of the RepViT Block is replaced with Dynamic Snake Convolution (DSConv). This structure employs re-parameterization techniques to eliminate the additional computational and storage costs associated with multi-branch architectures during inference. The CRVB-DSConvEMA module integrates the Efficient Multi-scale Attention (EMA) mechanism, ensuring a uniform distribution of spatial semantics across channels and sub-features. This design preserves multi-angle information while maintaining computational efficiency. It not only globally encodes information to adjust channel weights but also captures pixel-level relationships through cross-dimensional interactions. The improved CRVB-DSConvEMA structure is illustrated in Figure 8, and the RVB-DSConv module is depicted in Figure 9.

In Figure 8, when the input feature flows through the CRVB-DSConvEMA module, the preliminary features of the input $X\in R^{C\times H\times W}$ tensor are extracted, while the feature representation capability is enhanced through nonlinear activation. Subsequently, the divided two feature maps are fed into the RVB-DSConv block and the EMA block, respectively. The EMA attention mechanism captures long-range dependencies in the spatial dimension, and the local DSConv operation globally models the features, enhancing the global representation capability. Next, the features output from the multiple branches are concatenated or fused along the channel dimension to form the final feature tensor that combines multi-scale, local, and global information. Finally, the output from the previous layer undergoes a global EMA optimization, reintegrating information from different channels and spatial positions.

In Figure 9, the process begins with a 3×3 DSConv operation and a 1×1 DSConv operation for spatial feature extraction and fusion. This is followed by an SE layer (Squeeze-and-Excitation), which decouples the token mixer and channel mixer. Subsequently, through residual skip connections, the information is fused with the outputs of a 1×1 convolutional layer and a 1×1 projection layer, integrating channel and token mixer features. Finally, leveraging structural reparameterization, the computational load during the inference phase is significantly reduced compared to that during training.

#### 2.2.3. SPPF-DSConvEMA Module

The SPPF module in YOLOv8n has demonstrated its advantages in enhancing model performance through multi-scale feature fusion, especially in certain contexts. However, it must be acknowledged that the SPPF module may have limitations when dealing with variations in object scale and complex backgrounds. This is because, as the network deepens and frequent downsampling occurs, some positional information may be lost. Therefore, a fine-grained mechanism is needed to ensure that downsampling does not lead to the loss of fine-grained information.

**Figure 10:**
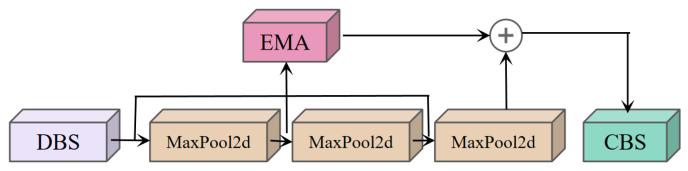
SPPF-DSConvEMA Module Structure Diagram.

To address the limitations of SPPF, the EMA attention mechanism is introduced to adaptively and dynamically adjust the weights in the feature map, ensuring the uniform distribution of spatial semantic features within each feature group. Meanwhile, DSConv is integrated into SPPF to ensure sufficient feature extraction from multiple perspectives before pooling. The specific structure of the proposed SPPF-DSConvEMA is shown in Figure 10, where the DBS module consists of DSConv, Batch Normalization (BatchNorm2d), and the activation function (SiLU).

In Figure 10, the tensor $X\in R^{C\times H\times W}$ (output from SPPF-DSConvEMA’s pooling branch) is divided into G sub-features along the channel dimension (Equation (10)) for multi-semantic learning. The grouped features are denoted as $X=\left[X_{0},X_{i},\ldots ,X_{i-1}\right],X_{i}\in R^{C//G\times W\times H}$ (usually G≪ C), ensures stable spatial grouping of feature semantics. After grouping, a three-branch parallel structure is formed to process features of different semantic levels respectively. Among them, two parallel branches are on the 1×1 path, while the other is on the 3×3 path. In the 1×1 convolutional path, dual encoding of channel features is achieved through one-dimensional global average pooling along the horizontal and vertical axes. In parallel, the 3×3 convolutional path employs a single 3×3 convolutional kernel to accomplish the fusion representation of multi-scale features. Here, the number of convolutional filters is independent of the batch dimension of the input data. This is because, in PyTorch, the parameter dimensions of a 2D convolutional kernel are defined as $\left[oup,\;inp,\;k,\;k\right]$, excluding the batch dimension. The oup parameter represents the spatial two-dimensional characteristics of the output feature map, inp denotes the two-dimensional plane of the input features, and k indicates the kernel size. Thus, by mapping the G groups to the batch dimension, the defined feature size becomes C//G×W×H, which serves as the tensor input. A dual-path Sigmoid activation function is applied to the decoupled feature vectors output by the 1×1 convolutional layer, performing nonlinear mapping to generate a two-dimensional probability distribution. Between the parallel 1×1 and 3×3 convolutional branches, a cross-dimensional heterogeneous feature interaction architecture is established. This architecture fuses dual-dimensional attention weights within each feature group through element-wise multiplication. Before multiplication, the dimensions of the two tensors are guaranteed to be identical because, prior to feature-wise concatenation and activation, global average pooling is applied for spatial feature encoding, transforming the branch outputs into the target dimensions. After generating the first spatial attention map, 2D global average pooling is similarly employed before fusion activation to directly encode the 3×3 branch and the global spatial information with a specific dimensional shape. This step subsequently yields a second spatial attention map, which fully retains precise spatial location information. In the final stage, the two generated spatial attention weights are combined to compute the output feature maps within each group. During this process, the Sigmoid function is applied. This method effectively captures pixel-level pairwise correlations and efficiently highlights the global contextual information embedded in all pixels.

#### 2.2.4. BiFPN Module

**Figure 11:**
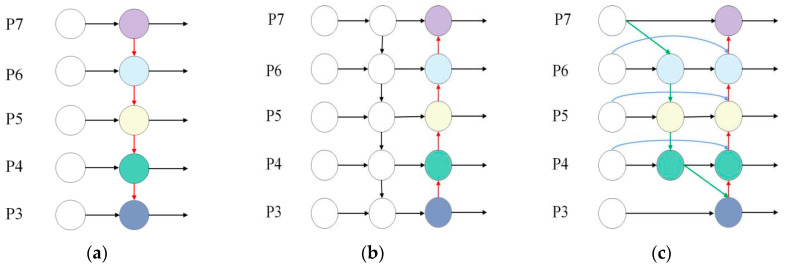
FPN, PANet, and BiFPN Structure Diagram. (**a**) FPN; (**b**) PANet; (**c**) BiFPN.

The neck network of YOLOv8 adopts a synergistic combination of the Path Aggregation Network (PAN) and the Feature Pyramid Network (FPN) architecture, as shown in Figure 11. The FPN framework effectively transfers deep feature information to shallower layers, enriching critical high-level insights. In contrast, the PAN structure facilitates the precise flow of localization data from shallow layers upward to feature-rich deep layers. This fusion creates the PANet [26] structure, which skillfully integrates shallow and deep features, significantly enhancing the model’s ability to recognize the most subtle details [27].

To significantly reduce the computational cost of the model and improve its practicality without compromising recognition accuracy, BiFPN is leveraged to achieve the research objective. This module enhances information flow between feature maps at different network levels through skip connections, allowing feature maps to retain more positional and detailed information, thereby helping the model focus better on key target information. Furthermore, it strengthens the original algorithm through more efficient bidirectional cross-scale connections and feature map fusion. Additionally, in terms of network topology, BiFPN integrates neural architecture search, a strategic approach designed to accommodate irregular feature network topologies under various task and resource constraints, thus offering greater flexibility in network design. Its well-crafted feature fusion and refinement operations not only improve accuracy but also successfully reduce computational complexity.

Taking the 6th layer of BiFPN as an example, its feature fusion is as follows:

(13) $$P_{6}^{td}=Conv\left(\frac{\omega_{1}\cdot P_{1}^{in}+\omega_{2}\cdot Resize\;\left(P_{7}^{in}\right)}{\omega_{1}+\omega_{2}+\varepsilon }\right)$$

(14) $$P_{6}^{out}=Conv\left(\frac{\omega_{1}^{\prime }\cdot P_{6}^{in}+\omega_{2}^{\prime }\cdot P_{6}^{td}+\omega_{3}^{\prime }\cdot Resize\;\left(P_{5}^{in}\right)}{\omega_{1}^{\prime }+\omega_{2}^{\prime }+\omega_{3}^{\prime }+\varepsilon }\right)$$

In Equations (13) and (14), $P_{6}^{td}$ represents the intermediate feature of the 6th layer in the top-down pathway, while $P_{6}^{out}$ denotes the output feature of the 6th layer. The feature fusion in other layers follows a similar pattern.

## 3. Results

### 3.1. Experimental Environment and Parameter Settings

The computer configuration used for training and testing in this experiment is shown in Table 3, and the main parameters of the specified program are listed in Table 4. Other parameters follow the default settings of the model.

### 3.2. Performance Evaluation Metrics

To evaluate the performance of YOLOv8n-RF, the following metrics were selected: Mean Average Precision (mAP), Model Size, Giga Floating-point Operations Per Second (GFLOPs), Accuracy, False Drop Rate (FDR), and Model Detection Time (MDT). The calculation formulas are as follows:

(15) $$Precision=\frac{T_{p}}{T_{p}+F_{p}}$$

(16) $$AP=\int_{0}^{1}Precision\left(t\right)dt$$

(17) $$mAP=\frac{\sum_{i=1}^{n}AP_{i}}{n}$$

(18) $$FLOPs=2\cdot H_{out}\cdot W_{out}\cdot C_{in}\cdot C_{out}\cdot K_{h}\cdot K_{w}$$

(19) $$FDR=\sum_{i=1}^{6}error_{i}$$

(20) $$MDT=\mathrm{MAX}\left\{time_{1},time_{2},\ldots ,time_{i}\right\},i=1,2,3,\ldots ,n$$

In Equation (18), $H_{out}$ and $W_{out}$ represent the height and width of the output feature map, $C_{in}$ and $C_{out}$ denote the number of input and output channels, $K_{in}$ and $K_{out}$ represent the height and width of the convolution kernel. In Equation (19), $error_{i}$ represents the probability of a certain category being falsely detected as the *i*-th category. In Equation (20), since YOLOv8 only provides the detection time ($time_{i}$) for each individual image during model prediction, the longest detection time observed in this study is taken as the value of DMT.

### 3.3. Experimental Analysis

#### 3.3.1. Ablation Experiment

To determine the importance of each proposed component and enhance the interpretability of the model, five sets of comparative experiments were conducted. Since this study involves testing on two datasets, the RemoteFinger dataset is abbreviated as RF, and the HaGRID dataset is abbreviated as HR for convenience. The experimental results are shown in Table 5.

In addition, to address false detections caused by incorrect gesture commands with features similar to correct ones, we put HaGRID’s untrained ‘rock’ category into the detection test and obtained the 1-FDR metric. Since the YOLOv8n model faced an untrained dataset, the experimental results showed that only the confusion matrix was valid, while other metrics (e.g., mAP) were 0. Therefore, the 1-FDR value was derived from the normalized confusion matrix. Furthermore, due to normalization, 1-FDR had a smaller error compared to FDR, so it was selected as the experimental metric.

As shown in Table 5, the baseline model YOLOv8n achieves a satisfactory mAP value when ignoring the parameters and GFLOPs metrics. However, additional experiments revealed that the presence of false detections still negatively impacted the user experience in real-world scenarios. After introducing the CRVB-DSConvEMA module into YOLOv8n, the model size was reduced by 1.32 M, and the false detection rate decreased by 7%, demonstrating the lightweight and efficient characteristics of CRVB-DSConv. Building upon this, the proposed SPPF-DSConvEMA module was incorporated. Since EMA and DSConv are not inherently lightweight but rather designed to enhance multi-angle feature extraction, the model size slightly increased by 0.01 M, and GFLOPs increased by 0.3. However, this adjustment further reduced the false detection rate. Finally, integrating BiFPN enabled efficient multi-scale feature fusion with minimal computational cost. Compared to the model before introducing BiFPN, the model size decreased by 1.18 M, GFLOPs decreased by 0.9, and the false detection rate was reduced by 5%.

Overall, compared to YOLOv8n, the proposed improvements result in a total reduction of 2.49 M in model size, a 1.7 decrease in GFLOPs, and a 22% reduction in the false detection rate, while the DMT increased by only 2.6 ms. Based on the experimental results presented in Table 5, it can be concluded that the proposed enhancement strategies significantly improve the detection performance of the baseline model YOLOv8n, demonstrating their effectiveness in reducing false gesture detections.

For convenience in future use, the models in Table 5 are assigned simplified names, with the simplification process detailed in Table 6.

**Figure 12:**
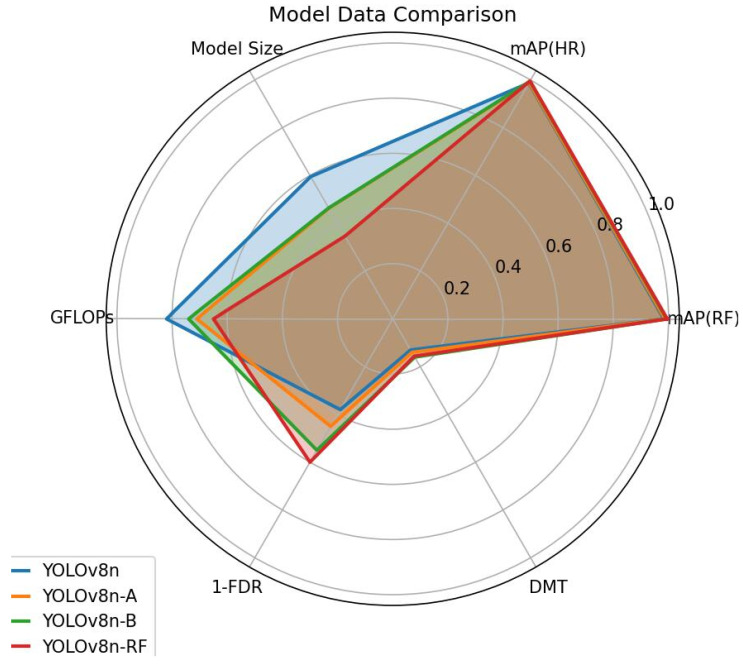
Comparison chart of experimental data for different models.

To illustrate the final improvement effect, the visualization of the data in Table 5 is shown in Figure 12.

**Figure 13:**
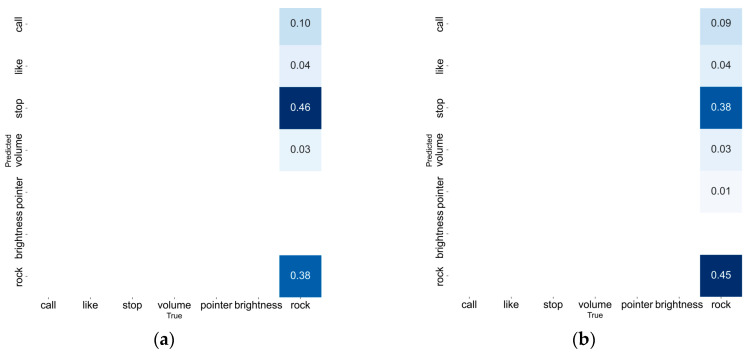

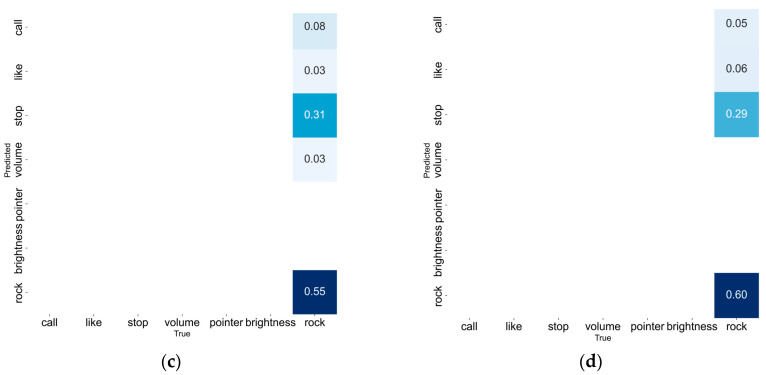
Normalized Confusion Matrix of False Detections for Each Module on the rock Category. (**a**) YOLOV8n; (**b**) YOLOv8n-A; (**c**) YOLOv8n-B; (**d**) YOLOv8n-RF.

Meanwhile, the normalized confusion matrix of false detections in this experiment is shown in Figure 13, and the detailed false detection data is presented in Table 7.

In Table 7, the sum of the FDR value and the (1-FDR) value for models YOLOv8n does not equal 1. This is because, after normalization, YOLOv8 retains only two significant digits in its results. Therefore, to minimize errors, (1-FDR) was used as the evaluation metric.

#### 3.3.2. Comparative Experiment

To compare the effectiveness of the detection model proposed in this paper, representative models with widely recognized detection efficiency in current research, SSD, MMDetectionv3, YOLOv5, YOLOv7, and YOLOv9, were selected for comparison and testing against the proposed model. All network models were trained and tested using the same dataset, with the number of training epochs set to 100. The same evaluation metrics were used as observation criteria, and the experimental results are shown in Table 8.

Table 8 shows that compared to other models, YOLOv8n-RF achieves the highest mAP and 1-FDR values, the lowest model size and GFLOPs, and its recall value is second only to the highest, with a difference of no more than 0.65%. Overall, the proposed model demonstrates outstanding performance across all metrics, combining lightweight characteristics with low false detection rates.

## 4. Discussion

**Figure 14:**
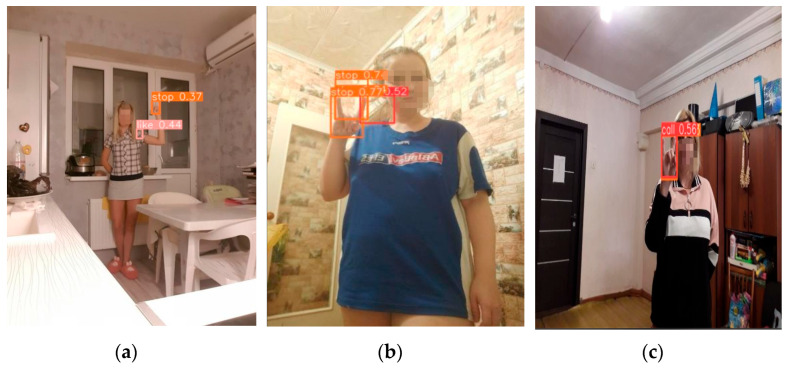
Example image of false detection cases. (**a**) Detection boxes without overlap; (**b**) Detection boxes partially overlapping; (**c**) Detection boxes completely overlapping.

As shown in Figure 14, the false detections observed in the experiment include cases of multiple detections: multiple detection boxes either not overlapping or overlapping with each other.

**Figure 15:**
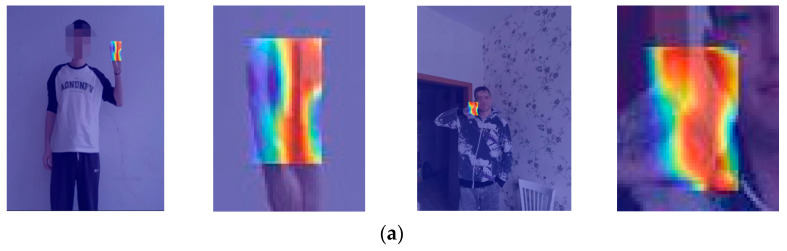

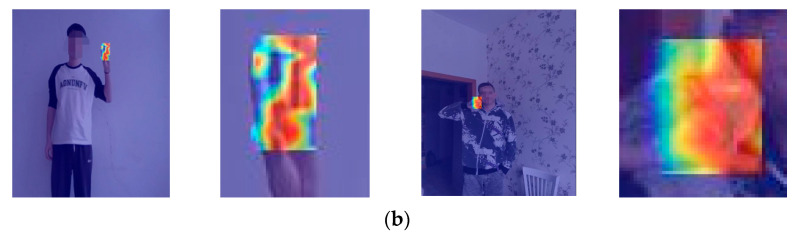
Comparison of heatmaps before and after model improvement. (**a**) YOLOv8n; (**b**) YOLOv8n-RF.

Furthermore, by computing the gradient of the feature map to generate a heatmap, we gain deeper insights into the model’s attention to relevant features during the detection process. The heatmaps generated before and after the model improvement are shown in Figure 15. In the heatmap, deeper red indicates higher attention, yellow represents moderate attention, and blue signifies minimal impact on image recognition, which the model considers as redundant information. It can be observed that the improved model focuses more on the shape features of fingers, with the main attention areas concentrated on the shape of the gesture, which is more beneficial for recognizing gesture commands.

**Figure 16:**
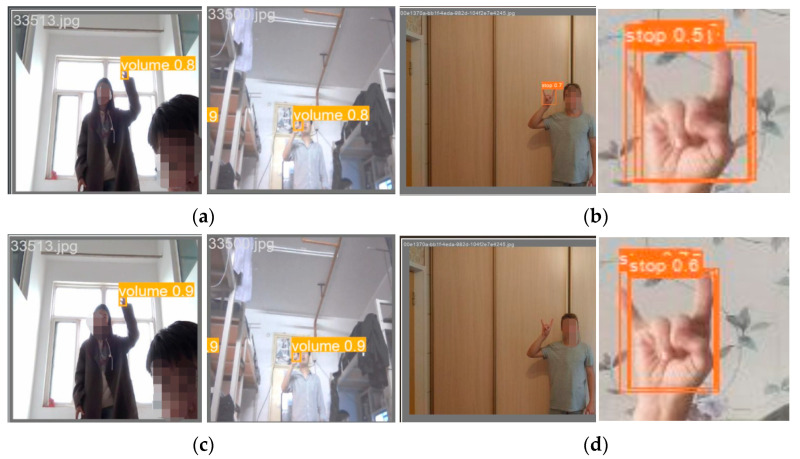
Comparison of detection performance between two algorithms. (**a**) YOLOv8n normal detection result image; (**b**) YOLOv8n false detection result image; (**c**) YOLOv8n-RF normal detection result image; (**d**) YOLOv8n-RF false detection result image.

For the comparison of detection performance in normal detection and false detection cases, refer to Figure 16. When detecting at close range, the results are almost identical, whereas at long distances, there are noticeable differences. The first row shows the detection results of YOLOv8, while the second row displays the results of the improved model. Compared to the YOLOv8 baseline model, the proposed algorithm in this study demonstrated better detection performance in our experiments. Specifically, in long-distance detection, the confidence score was higher than that of the baseline model. Regarding false detection, the proposed model correctly detected the object in the first example, while its performance was slightly inferior to the original model in the second example. This is because the proposed model does not entirely eliminate false detections. However, considering the overall detection performance, the improved algorithm significantly outperformed the original algorithm.

**Figure 17:**
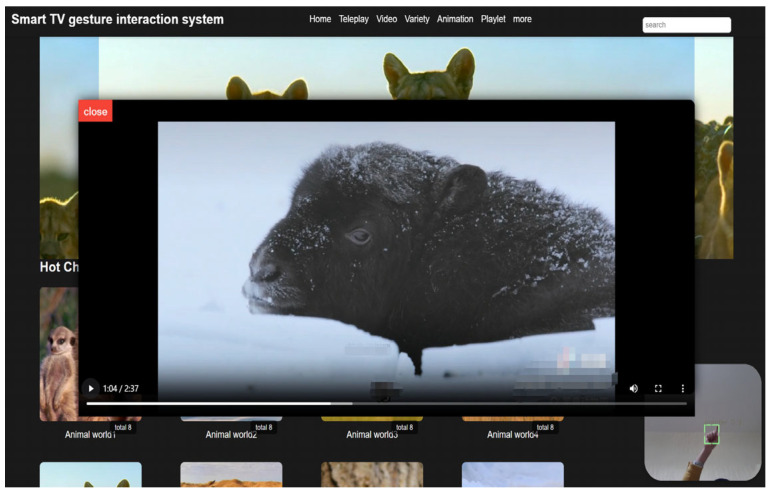
Example of remote control finger interaction tested with YOLOv8n-RF.

In the final stage of this study, the dynamic gesture detection results of YOLOv8n-RF were mapped into remote control finger commands, and a compatible system was developed accordingly. Validation on this system demonstrated seamless interaction, further confirming the effectiveness of the algorithm. An experimental example is shown in Figure 17.

## 5. Conclusions

To address the issues of gesture commands being easily misrecognized and the high complexity of the model, this paper proposes the YOLOv8n-RF dynamic remote finger detection algorithm. This algorithm reduces the false detection rate of gesture command detection while achieving high detection performance with lower computational complexity. By embedding the CRVB-DSConvEMA module into the feature extraction network, the model effectively preserves fine morphological characteristics from multiple angles, reducing false detections while maintaining low computational complexity. Additionally, the introduction of the SPPF-DSConvEMA module allows adaptive weight adjustment during downsampling, preserving semantic information from multiple angles without losing important details. Finally, the BiFPN module is incorporated to further lightweight the model without compromising its performance.

Experiments were conducted on a custom Remote Finger dataset, the publicly available HaGRID dataset, and additional “rock” class data that were not used in training, covering a total of seven categories. Among them, the ‘rock’ class did not belong to the six predefined categories for training purposes and was solely used for false detection testing. The results showed that, compared to the original YOLOv8n, the YOLOv8n-RF algorithm achieved improvements of 1.23% and 0.84% in mAP along with 0.78% and 0.38% gains in recall on the two datasets, respectively, reduced model size by 2.49 M, decreased GFLOPs by 1.7, and lowered the false detection rate by 22%, while the DMT increased by only 2.6 ms. The YOLOv8n-RF algorithm effectively reduces false detections, thereby minimizing erroneous control actions. Additionally, it exhibits a certain level of generalization, making it suitable for real-world smart TV gesture interaction applications based on monocular cameras.

## Figures and Tables

**Table 1:** RemoteFinger Dataset Description.

Dataset	Volume	Pointer	Brightness	Total (Images)
Remote Finger 1.0	2100	2100	2100	6300
Remote Finger	2080	2080	2039	6199

**Table 2:** Filtered HaGRID Dataset Description Table.

Dataset	Call	Like	Stop	Total (Images)
HaGRID	1988	1988	1988	5994

**Table 3:** Experimental Environment.

Name	Version
CPU	Intel(R) Xeon(R) Silver 4214R
GPU	RTX 3080 Ti(12 GB) *1
OS	Ubuntu 18.04
PyTorch	1.13.0
Python	3.9.18
CUDA	11.7
OpenCV	4.8.1.78

**Table 4:** Main Parameters of the Program.

Name	Version
epoch	100
imgsz	640
batch	32
workers	8
device	0

**Table 5:** Comparison of experimental results before and after model improvement.

CRVB-DSCovnEMA	SPPF-DSConvEMA	BiFPN	mAP (%) RF	mAP (%) HR	Model Size (M)	GFLOPs	1-FDR Rock	DMT (ms) RF
-	-	-	98.30	98.73	5.95	8.2	38	13.0
√	-	-	98.70	98.92	4.63	7.1	45	14.0
√	√	-	99.30	99.43	4.64	7.4	55	16.0
√	√	√	99.53	99.57	3.46	6.5	60	15.6

**Table 6:** Table of Simplified Module Names.

Name	Model
YOLOv8n-A	CRVB-DSConvEMA
YOLOv8n-B	CRVB-DSConvEMA+SPPF-DSConvEMA
YOLOv8n-RF	CRVB-DSConvEMA+SPPF-DSConvEMA+BiFPN

**Table 7:** The situation where each module misclassifies “rock” into six different categories.

Model	Call	Like	Stop	Volume	Pointer	Brightness	FDR	1-FDR
YOLOv8n	0.10	0.04	0.46	0.03	-	-	0.63	0.38
YOLOv8n-A	0.09	0.04	0.38	0.03	0.01	-	0.55	0.45
YOLOv8n-B	0.08	0.03	0.31	0.03	-	-	0.45	0.55
YOLOv8n-RF	0.05	0.06	0.29	-	-	-	0.40	0.60

**Table 8:** Performance metric data of various object detection models.

Model	mAP (%) RF	Recall (%) RF	mAP (%) HR	Recall (%) HR	Model Size (M)	GFLOPs	1-FDR (%) Rock
SSD	21.13	-	88.33	-	186	-	20
MMDectionv3	47.43	58.33	65.00	64.00	59.9	-	40
YOLOv5	98.67	98.50	99.33	97.50	5.01	7.1	28
YOLOv6	97.43	96.83	99.20	98.33	8.28	11.8	20
YOLOv7	98.70	99.23	99.00	98.67	11.7	13.2	39
YOLOv8	98.30	98.20	98.73	98.50	5.95	8.2	38
YOLOv9	98.53	97.43	99.37	99.53	5.80	11.0	30
YOLOv8n-RF	99.53	98.98	99.57	98.88	3.46	6.5	60

## Data Availability

The original contributions presented in the study are included in the article.
